# Differential synovial fluid white blood cell count for the diagnosis of chronic peri-prosthetic joint infection – a systematic review and meta-analysis

**DOI:** 10.5194/jbji-10-165-2025

**Published:** 2025-05-14

**Authors:** Marta Sabater-Martos, Martin Clauss, Ana Ribau, Ricardo Sousa, on behalf of the Leukocyte Count Synovial Fluid working group for the Unified PJI definition task force

**Affiliations:** 1 Orthopedic and Traumatology Department, Clínic Barcelona. Carrer Villarroel 170, 08036 Barcelona, Spain; 2 Center for Musculoskeletal Infections (ZMSI), University Hospital Basel, Basel, Switzerland; 3 Department for Orthopaedics and Trauma Surgery, University Hospital Basel, Basel, Switzerland; 4 Unidade Local de Sáude do Médio Ave, Famalicao, Portugal; 5 Porto Bone and Joint Infection Group (GRIP), ULS Santo António – Porto and CUF hospitals, Porto and Lisbon, Portugal; ➕ A full list of authors appears at the end of the paper

## Abstract

**Introduction**: Peri-prosthetic joint infection (PJI) is a significant complication of arthroplasty, lacking a single gold standard diagnostic test. Synovial fluid white blood cell (WBC) count and polymorphonuclear neutrophil (PMN) proportion are widely used diagnostic tools, but their optimal cutoffs remain unclear, particularly for chronic PJI. **Material and methods**: This systematic review and meta-analysis included 74 studies published between 2000 and 2024. Data on diagnostic performance (sensitivity, specificity, and diagnostic odds ratios – DORs) of WBC count and PMN proportions were analysed. Sub-group analyses and heterogeneity assessments were performed, and optimal cutoffs for diagnostic accuracy were identified. **Results**: The meta-analysis revealed a WBC count summary DOR of 58.38 (95 % CI – confidence interval: 48.48–70.32) with an area under the curve (AUC) of the summarized receiver operating characteristic curve of 0.952. The PMN proportion showed a DOR of 43.17 (95 % CI: 35.31–52.79) and an AUC of 0.941. Optimal diagnostic thresholds for chronic PJI were WBC count > 2600 cells per microlitre and PMN > 70 %. Rule-in thresholds (specificity >95 %) were WBC count ≥ 3000 cells per microlitre and PMN ≥ 75 %, while rule-out thresholds (sensitivity > 95 %) were WBC count ≤ 1500 cells per microlitre and PMN ≤ 65 %. Confounding conditions such as fractures, inflammatory arthritis, and metal-related reactions reduced test accuracy. **Conclusions**: Synovial fluid analysis remains a critical diagnostic tool for chronic PJI. Thresholds of WBC count < 1500 and >3000 cells per microlitre and PMN < 65 % and >75 % provide reliable negative and positive predictive values. A standardized diagnostic framework is essential for addressing remaining controversies and ensuring consistent interpretation across clinical settings.

## 1 Introduction

Peri-prosthetic joint infection (PJI) is a potentially dismal complication of total joint arthroplasty. As the expected number of arthroplasties is rising worldwide, so, too, is the burden of the small but consistent infection rate (Premkumar et al., 2021; Rupp et al., 2020). In fact, infection is a leading cause of total joint replacement failure, and it is critical to accurately diagnose it, as failure to do so will hamper treatment success (Sousa et al., 2023). Still, there is no available gold standard diagnostic test on its own. The definition of PJI thus relies on the combined interpretation of multiple different tests (Osmon et al., 2013; Parvizi and Gehrke, 2014; Shohat et al., 2019; Sousa et al., 2023; Workgroup Convened by the Musculoskeletal Infection Society, 2011).

Synovial fluid differential white blood cell (WBC) count is a widely available and rather inexpensive test that has long been recognized as a critical part of the preoperative workup before revision arthroplasty. Its accuracy for the diagnosis of PJI in the setting of chronically painful joints has been studied extensively and gives consistently good results.

There are, nevertheless, open questions. First, different proposed cutoffs exist due to variations in PJI definitions and joint-specific studies. Second, conditions such as peri-prosthetic fractures, inflammatory arthritis, crystal arthropathy, or metal-ion-related tissue reactions can elevate WBC counts irrespective of infection, affecting accuracy. Third, the role of differential WBC count in acute postoperative PJI remains less clear due to post-surgical inflammation.

This systematic review aimed to (1) determine the overall diagnostic accuracy of synovial WBC counts and polymorphonuclear neutrophil (PMN) proportions for chronic PJI, (2) establish optimal interpretation cutoffs, and (3) evaluate test performances under confounding conditions.

## 2 Material and methods

This systematic review adheres to PRISMA of Diagnostic Test Accuracy Studies (PRISMA-DTA) (McInnes et al., 2018).

### 2.1 Search strategy

A computer-aided search on MEDLINE and EMBASE (1 January 2000–1 February 2024) identified studies evaluating synovial WBC count and PMN proportion for PJI diagnosis. Reference lists of included articles were also reviewed. The search strategy combined terms related to arthroplasty infection, synovial fluid diagnosis, and diagnostic accuracy (see Table S1 in the Supplement).

### 2.2 Inclusion and exclusion criteria

Studies were included if they evaluated synovial fluid WBC count and/or PMN proportion in suspected PJI cases, provided diagnostic performance data, were conducted on humans ≥18 years old, and were published in English between 2000 and 2024. Only studies reporting results on chronic PJI were included. We excluded studies on infections unrelated to PJI, case reports, animal studies, and reviews.

**Figure 1:**
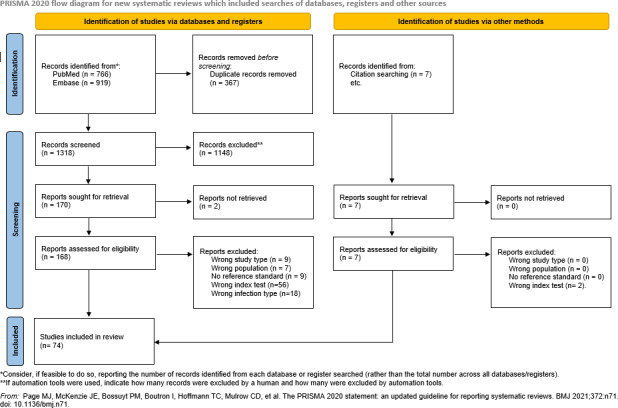
PRISMA flow diagram.

Duplicates were removed using Mendeley. Screening was performed with Rayyan software by two independent reviewers per article, first by title or abstract and then by full text. Disagreements were resolved by consensus. The study selection process is shown in Fig. 1 (Page et al., 2021).

### 2.3 Data extraction

Data were extracted in duplicate, resolving discrepancies by consensus. Collected data included study characteristics, diagnostic criteria, infection type, patient characteristics (e.g. inflammatory diseases, fractures, metallosis, or inflammatory arthritis), WBC count and PMN cutoffs, sensitivity, specificity, predictive values, area under the curve (AUC), accuracy, and Youden index. True positive, true negative, false positive, and false negative values were calculated.

### 2.4 Risk of bias and quality assessment

Two independent investigators per article assessed the risk of bias and applicability of each included study by using the Quality Assessment of Diagnostic Accuracy Studies (QUADAS-2) (Whiting et al., 2011) tool, with ratings of “low”, “high”, or “unclear”. Discrepancies were resolved by consensus.

Quality was assessed by two independent investigators per article with the Grading of Recommendations Assessment, Development and Evaluation (GRADE) tool (Schünemann et al., 2023). This tool is used to evaluate the quality of the evidence and the strength of the recommendations. Studies were classified as “high (A)”, “moderate (B)”, “low (C)”, or “very low (D)” quality.

### 2.5 Information synthesis and statistical analysis

With the information extracted, we calculated the diagnostic odds ratio (DOR) for every study. Although sensitivity and specificity are commonly used in diagnostic test research due to their clinical relevance, these metrics can introduce a threshold effect when multiple studies are compared. Although the DOR has limited direct clinical application, it is particularly useful in systematic reviews of diagnostic accuracy because it reflects the overall effectiveness of a diagnostic test (Arias and Molina, 2015; De Sousa et al., 2009). We used DORs along with 95 % confidence intervals (CIs) and summarized receiver operating characteristic (sROC) curves as part of the meta-analysis (De Sousa et al., 2009). The analyses were conducted by using a random-effects model and the DerSimonian–Laird approach. In addition, a sub-group analysis was performed based on the joint involved (hip or knee).

Heterogeneity in the reported sensitivities and specificities was assessed by using Higgins' $I^{2}$ statistic. All statistical analyses were performed with Jamovi (version 2.2.5) and Meta-disc (versions 1–4).

### 2.6 Cutoff determination

To determine interpretation cutoff values, we used only those articles that calculated their own ideal cutoff based on their data and excluded all papers that used pre-determined cutoffs. We excluded from this analysis studies that focused on patients with confounding circumstances such as fractures or dislocation, inflammatory arthritis, crystal arthropathy, or adverse local tissue reactions due to metal ions.

We used three different data interpretation strategies: (1) the “optimal” cutoff based on the best combination of specificity and sensitivity, (2) a “rule-in” cutoff that focused on optimizing specificity, and (3) a “rule-out” cutoff that focused on optimizing sensitivity.

We used the Youden index and extracted cutoffs and presented them in a scatterplot diagram. The optimal cutoff was determined by using median values. We obtained optimal cutoffs for combined total knee arthroplasty (TKA) and total hip arthroplasty (THA) as well as for TKA and THA individually.

To estimate an ideal rule-in/rule-out threshold, we decided to use only specificity or sensitivity values of >95 %. We used specificity or sensitivity vs. extracted cutoffs and presented them in a scatterplot diagram, and the rule-in/rule-out cutoff was determined using median values. We obtained the rule-in/rule-out value for the combined TKA and THA. There were not enough data to extract a rule-in value for TKA and THA individually.

We performed a post hoc sub-analysis by sub-dividing groups between the modern or more sensitive PJI definition criteria (International Consensus Meeting ICM, European Bone and Joint Infection Society EBJIS, and Infectious Disease Society of America IDSA) and the old PJI definition criteria (Musculoskeletal Infection Society MSIS and clinical outcome) to analyse the sensitivity and specificity cutoff values of these two groups. Information concerning other joints (e.g. shoulders, elbows, hemi-hips, uni-knees, or ankles) was too scarce to merit meaningful calculations.

## 3 Results

### 3.1 Search results

The search identified 1685 records. After removing duplicates, 1318 studies underwent title and abstract screening. Of 170 full-text studies assessed, 96 were excluded for reasons such as wrong study type (9), incorrect population (7), unspecified reference standard (9), different index test (56), or lack of chronic PJI separation (18). Five additional studies were identified through bibliography review, yielding 74 included studies (Fig. 1).

### 3.2 Risk of bias and quality assessment

**Figure 2:**
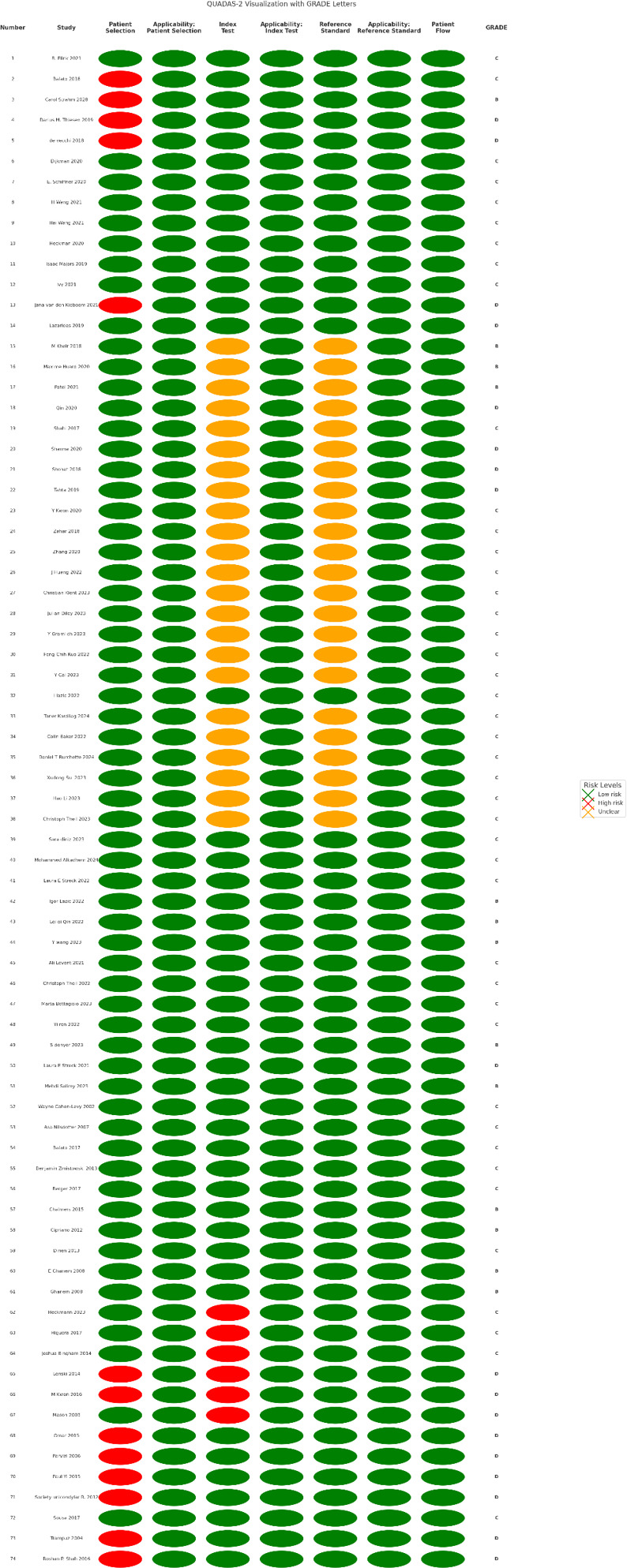
QUADAS-2 and GRADE assessment.

Results from QUADAS-2 (Whiting et al., 2011) are presented in Fig. 2. Most studies had a high risk of bias but good applicability, largely due to retrospective study designs. Patient selection was unclear in more than 10 studies, while index test and reference standard issues were found in more than 20 studies.

GRADE assessment classified 13 studies (17.6 %) as B, 43 (58.1 %) as C, and 18 (24.3 %) as D (Fig. 2). Because of the retrospective nature of the majority of the papers, more than 50 % of the studies included were classified as GRADE C.

### 3.3 Characteristics of the studies

We included 74 studies (Table 1). The publication years ranged from 2003 to 2024, the year with the highest publication rate being 2023 with 13 papers. Twenty-six papers were prospective cohorts (35.1 %) and 48 were retrospective cohorts (64.9 %), covering 18 960 patients (19 to 4462). Papers used different PJI reference standards: 14 used clinical definitions (18.9 %), 3 the IDSA 2013 definition (4.1 %), 35 the MSIS 2013 definition (47.3 %), 17 the ICM 2018 definition (23 %), and 4 the EBJIS 2021 definition (5.4 %). One paper combined MSIS 2013 and ICM 2018.

**Table 1:** General characteristics of primary studies.

Author	Year	Type of study	Infected	Joints	PJI	Special condition	WBC	Sensitivity	PMN	Sensitivity	Cutoff	Reporting
			patients/		reference		count	and	cutoff	and	determination	funds
			total		standard		cutoff	specificity		specificity		
Bernd Fink (Fink et al.,	2021	Prospective	112/434	Hips and	ICM 2018	No	1400	90.2 %/91.9 %	NA	NA	Youden	NM
2021)				knees			500	98.8 %/91 %			Youden	
							1000	93.8 %/92.9 %			Youden	
							1500	90.2 %/95.7 %			Youden	
							2000	86.6 %/91.2 %			Youden	
							3000	80.6 %/94.9 %			Youden	
Giovanni Balato (Balato et	2018	Retrospective	31/167	Knees	MSIS	No	3000	80.6 %/91.2 %	80 %	83.9 %/94.9 %	Youden	NM
al., 2018a)												
Carol Strahm	2018	Retrospective	13/19	Shoulders	IDSA	No	12 200	92 %/100 %	54 %	100 %/75 %	Youden	NM
(Strahm et												
al., 2018)												
Darius M. Thiesen	2019	Retrospective	2/31	Ankles	MSIS	No	NA	100 %/37.5 %	NA	100 %/77.8 %	Youden	No
(Thiesen et al.,												funding
2019)												
Elena De Vechhi (De	2018	Retrospective	32/66	Hips and	MSIS	No	3000	93.7 %/91.2 %	NA	NA	Pre-determined	NM
Vecchi et al., 2018)				knees			1600	100 %/82.3 %			TWC	
Casper Dijkman (Dijkman	2020	Retrospective	13/89	Hips and	MSIS	No	2575	92 %/84 %	NA	NA	Youden	NM
et al., 2020)				knees			1865	100 %/97 %			Youden	
Erik Schiffner	2020	Retrospective	32/149	Knees	MSIS	No	3000	84 %/96 %	65 %	84 %/94 %	Pre-determined	NM
(Schiffner et al.,												
2020)												
Hai Wang (Wang et	2021	Prospective	37/93	Hips and knees	MSIS	No	NA	NA	69.96 %	94.6 %/92.8 %	Youden	NM
al., 2021b)												
Hai Wang (Wang et	2021	Prospective	39/97	Hips and	MSIS	No	NA	NA	69.8 %	84.6 %/74.1 %	Youden	NM
al., 2021a)				knees								
Heckman	2020	Prospective	16/78	Hips and	MSIS	No	3000	100 %/100 %	80 %	81.3 %/96.8 %	Pre-determined	NM
Nathanael Heckmann				knees			(pre-		(pre-			
(Heckmann et							lavage)		lavage)			
al., 2020)							3000	62.5 %/98.4 %	80 %	75 %/95.2 %	Pre-determined	
							(post-		(post-			
							lavage)		lavage)			
Isaac Majors (Majors	2019	Prospective	32/59	Knees	MSIS	No	1804	%/81 %	86 %	82.4 %/73.7 %	Youden	NM
and Jagadale, 2019)												
Ivy (Ivy et al., 2021)	2021	Prospective	18/99	Knees	MSIS	No	1700	83.3 %/93.8 %	65 %	83.3 %/94.9 %	Pre-determined	NM
				Hips			3000	83.3 %/93.8 %	80 %	83.3 %/94.9 %	Pre-determined	

**Table 1:** Continued.

Author	Year	Type of study	Infected	Joint	PJI	Special condition	WBC	Sensitivity	PMN	Sensitivity	Cutoff	Reporting
			patients/		reference		count	and	cutoff	and	determination	funds
			total		standard		cutoff	specificity		specificity		
Jana van den Kieboom (van	2021	Retrospective	41/144	Hips and	MSIS	Fracture	3000	87 %/77.9 %	80 %	79 %/63.2 %	Pre-determined	NM
den Kieboom et al.,				knees			4552	86.4 %/85.3 %	79.5 %	73.7 %/63.2 %	Q point	
2021)												
Alexander Lazarides	2019	Retrospective	16/87	Hips and	MSIS	Immunosuppression	1293	93.3 %/84.4 %	65 %	81.3 %/87.3 %	Youden	Yes
(Lazarides et al.,				knees								
2019)												
Michael M. Kheir (Kheir et	2018	Retrospective	549/1202	Hips and	ICM 2018	No	2659	85 %/93 %	64.5 %	91 %/86 %	Youden	No
al., 2018)				knees			3000	83 %/94 %	80 %	78 %/93 %	Pre-determined	funding
Maxime Huard (Huard et	2020	Prospective	41/138	Hips and	MSIS	No	3000	87.7 %/95.7 %	NA	NA	Pre-determined	NM
al., 2020)			50/138	knees	IDSA		3000	70 %/94.2 %			Pre-determined	
			68/138		EBJIS		3000	55.8 %/97 %			Pre-determined	
			35/138		Positive		881	80 %/91 %			Youden	
					culture							
					Positive		3077	91 %/85 %			Youden	
					culture							
Vikas V. Patel (Patel et al.,	2021	Retrospective	20/87	Shoulders	ICM 2018	No	3000	30 %/100 %	80 %	20 %/100 %	Pre-determined	NM
2021)												
Leilei Qin (Qin et al.,	2020	Prospective	25/50	Hips and	MSIS	No	NA	NA	69.79 %	92 %/80 %	Youden	NM
2020)				knees								
Alisina Shahi (Shahi et al.,	2017	Retrospective	822/4462	Hips and	MSIS	No	3000	85.8 %/83 %	80 %	85.8 %/80.8 %	Pre-determined	NM
2017)				knees								
Katyayini Sharma (Sharma et	2020	Retrospective	50/107	Hips and	MSIS	No	1100	89 %/98 %	72 %	92 %/91 %	Pre-determined	NM
al., 2020)				knees								
Noam Shohat (Shohat et	2018	Retrospective	567/1220	Hips and	MSIS	No	3000	81.8 %/95.1 %	80 %	77.7 %/94.3 %	Pre-determined	NM
al., 2018)				knees		No	2533	83.4 %/93.3 %	73 %	86.8 %/89.9 %	Youden	
						Inflammation	3000	88.2 %/78.6 %	80 %	93.7 %/84.6 %	Pre-determined	
						Inflammation	2683	91.2 %/78.6 %	72 %	100 %/82.7 %	Youden	
Mesut Tahta (Tahta et al.,	2019	Prospective	17/38	Knees	MSIS	Inflammation	2347	84.6 %/76.4 %	76.7 %	79.2 %/90.3 %	Youden	NM
2019)												
Young-Min Kwon (Kwon et	2020	Retrospective	11/89	Hips	MSIS	ATRLs	2144	93 %/84 %	84 %	84 %/86 %	Youden	No
al., 2020)							13 796	100 %/100 %	82 %	93 %/82 %	Youden	funding
Akos Zahar (Zahar et al.,	2018	Retrospective	134/337	Hips and	MSIS	No	2582	80.6 %/85.2 %	66.1 %	80.6 %/83.3 %	Youden	NM
2018)				knees								
							1630	83.5 %/82.2 %	60.5 %	80.3 %/77.1 %	Youden	
				Hips and			3063	78.1 %/80 %	66.1 %	82.2 %/82.4 %	Youden	
				knees								

**Table 1:** Continued.

Author	Year	Type of study	Infected	Joints	PJI	Special condition	WBC	Sensitivity	PMN	Sensitivity	Cutoff	Reporting
			patients/		reference		count	and	cutoff	and	determination	funds
			total		standard		cutoff	specificity		specificity		
Zeyu Zhang (Zhang et al.,	2020	Retrospective	21/63	Hips and	MSIS	No	3000	85.7 %/83.3 %	65 %	90.5 %/85.7 %	Youden	NM
2020)				knees								
Jiaxing Huang (Huang et	2022	Prospective	43/99	Hips and	MSIS	No	3000	85.7 %/83.3 %	75 %	95.3 %/78.6 %	Youden	NM
al., 2022)				knees								
Christian Klemt (Klemt et	2023	Retrospective	191/464	Hips	ICM 2018	No	3000	86.9 %/79.1 %	80 %	87.9 %/78.7 %	Pre-determined	NM
al., 2023)												
Julian E. Dilley (Dilley et	2023	Retrospective	321/730	Hips and	MSIS	No	5600	71.8 %/85.6 %	82 %	81 %/77.5 %	Youden	NM
al., 2023)				knees			3000	76 %/80.2 %	80 %	82.2 %/76 %	Pre-determined	
Y. Gramlich	2023	Prospective	111/405	Hips,	ICM 2018	No	2478.9	87.7 %/88.1 %	67 %	86 %/88.8 %	Youden	NM
Yves Gramlich				knees,			1500	91.5 %/75 %	65 %	86 %/88.8 %		
(Gramlich et al.,				and			1750	89.6 %/78.7 %	70 %	82.6 %/90 %		
2023)				shoulders			2000	89.6 %/83.4 %				
				Knees			2500	86.6 %/88.1 %				
				Hips			3000	82.1 %/91 %	80 %	66.3 %/96.5 %	Pre-determined	
							3085.7	86.7 %/91.8 %	67.01 %	87.5 %/88.2 %	Youden	
							2267.8	87.5 %/83.5 %	67.04 %	81.5 %/94.1 %	Youden	
Feng Chih Kuo (Kuo	2022	Retrospective	42/76	Hips and	ICM 2018	No	3000	68 %/94 %	70 %	71 %/84 %	Pre-determined	No
et al., 2022)				knees								funding
Yuanquing Cai (Cai et al.,	2023	Retrospective	46/74	Hips and	MSIS	No	3000	63 %/74 %	80 %	78.2 %/71.4 %	Pre-determined	NM
2023)				knees								
Igor Lazic (Lazic et al.,	2022	Prospective	17/32	Hips and	EBJIS	Fracture	6130	41 %/100 %	79.5 %	71 %/100 %	Youden	NM
2022a)				knees								
Taner Karlidag	2024	Retrospective	50/144	Knees	ICM 2018	No	2553.5	92 %/91.4 %	81.7 %	88 %/93.6 %	Youden	No
(Karlidag et al.,			59/116	Hips			3512.5	83 %/85.9 %	83.6 %	86.4 %/93.6 %	Youden	funding
2024)												
Colin M. Baker (Baker et	2022	Retrospective	194/621	Hips and	ICM 2018	No	3000	85.7 %/100 %	80 %	92.6 %/96 %	Pre-determined	NM
al., 2022)				knees								
			162/520	Knees			3000	92 %/99 %	80 %	86.4 %/99.2 %	Pre-determined	
			32/68	Hips			3000	92.8 %/98.5 %	80 %	85.3 %/99.4 %	Pre-determined	
Daniel Timothy Burchette	2024	Retrospective	185/362	Hips and	ICM 2018	No	2581	74.3 %/88 %	75.4 %	68.8 %/97.6 %	Youden	NM
(Burchette et al.,					knees							
2024)				111/195	Hips		2373	81 %/77.8 %	73.4 %	72.2 %/98.1 %	Youden	
				73/165	Knees		4058	66.7 %/97.2 %	75.4 %	64.8 %/97.2 %	Youden	
Xudong Su (Su et al.,	2023	Retrospective	30/60	Hips and	MSIS	No	NA	NA	51.1 %	96.7 %/73.3 %	Youden	NM
2023)				knees								
Hao Li (Li et al.,	2023	Retrospective	48/90	Hips and	ICM 2018	No	3000	74 %/94.4 %	70 %	79.6 %/79.6 %	Pre-determined	NM
2023)				knees								

**Table 1:** Continued.

Author	Year	Type of study	Infected	Joints	PJI	Special condition	WBC	Sensitivity	PMN	Sensitivity	Cutoff	Reporting
			patients/		reference		count	and	cutoff	and	determination	funds
			total		standard		cutoff	specificity		specificity		
Cristoph Theil (Theil et al.,	2023	Retrospective	55/108	Knees	MSIS	ALTRs	1200	94.5 %/75.5 %	63 %	85.5 %/73.6 %	Youden	NM
2024)							1500	89.1 %/79.2 %	65 %	85.5 %/73.6 %	Pre-determined	
									70 %	76.3 %/77.4 %	Pre-determined	
							3000	70.1 %/94.3 %	80 %	60 %/81.1 %	Pre-determined	
Sara Elisa Diniz	2023	Retrospective	36/102	Hips and	EBJIS	No	1470	91.7 %/92.4 %	62.5 %	88.9 %/83.9 %	Youden	NM
(Diniz et al.,				knees			2645	77.8 %/98.5 %	64.4 %	86.1 %/88.7 %	Youden	
2023)							3280	77.8 %/100 %	79.5 %	75 %/98.4 %	Youden	
Mohammed F. Alkadhem	2024	Retrospective	19/137	Hips and	EBJIS	No	1500	74 %/71 %	65 %	89 %/78 %	Pre-determined	NM
(Alkadhem et al.,				knees			3000	75 %/86 %	80 %	89 %/85 %	Pre-determined	
2024)				Knees			3000	100 %/87 %	80 %	100 %/85 %	Pre-determined	
				Hips			3000	58 %/84 %	80 %	91 %/84 %	Pre-determined	
Sara Elisa Diniz (Streck	2022	Retrospective	21/35	Shoulders	ICM 2018	No	700	85.7 %/100 %	NA	NA	Youden	NM
et al., 2022b)												
Igor Lazic (Lazic et al.,	2022	Prospective	14/30	Hips and	EBJIS	Fracture	4550	40 %/100 %	79 %	30 %/100 %	Youden	NM
2022b)				knees								
Leilei Qin (Qin et al.,	2022	Prospective	35/70	Hips and	MSIS	Inflammation	NA	NA	69.8 %	88.6 %/80 %	Youden	NM
2022)				knees								
Yulai Wang (Wang et	2023	Retrospective	164/348	Hips and	MSIS	No	2673	80.5 %/96.4 %	62 %	92.8 %/92.9 %	Youden	Yes
al., 2023)				knees		Inflammation	3654	80.5 %/94.4 %	65.9 %	85.4 %/77.8 %	Youden	
Ali Levent (Levent et	2021	Retrospective	109/260	Hips and	ICM 2018	No	3000	87.2 %/88.1 %	70 %	89.9 %/84.1 %	Pre-determined	No
al., 2021)				knees			2950	88.1 %/88.1 %	77.8 %	89.9 %/91.4 %	Youden	funding
Cristoph Theil (Theil et al.,	2022	Prospective	30/92	Hips and	ICM 2018	No	3000	90 %/88 %	80 %	73 %/98 %	Pre-determined	NM
2022)				knees								
Martra Bottagisio	2023	Prospective	12/55	Hips and	ICM 2018	No	6740	85.7 %/97.1 %	85 %	85.7 %/97.1 %	Youden	NM
(Bottagisio et al.,				knees								
2023)												
Yi Ren (Ren et al.,	2020	Retrospective	63/NA	Hips and	Cultures	Inflammation	1948	83.3 %/72.7 %	85.3 %	83.3 %/73 %	Youden	NM
2022)				knees								
Steven Denyer (Denyer	2023	Retrospective	124/283	Knees	IDSA	No	3000	89.9 %/89.7 %	80 %	89.5 %/100 %	Pre-determined	NM
et al., 2023)			122/289	Hips		No	3000	82.5 %/80 %	80 %	82.5 %/62.5 %	Pre-determined	
Laura Elisa Streck (Streck	2021	Retrospective	15/31	Shoulders	IDSA	No	2800	86.7 %/87.5 %	NA	NA	Youden	NM
et al., 2022a)												
Mehdi S. Salymi (Salimy	2023	Retrospective	116/207	Hips and	ICM 2018	Antibiotic	2339	95.2 %/93.3 %	83 %	90.5 %/80 %	Youden	No
et al., 2023)				knees								funding
Wayne B. Cohen-Levy	2002	Retrospective	20/109	Knees	MSIS +	No	2695	94 %/90 %	52.5 %	94 %/93 %	Youden	No
(Cohen-Levy et al.,					ICM 2018							funding
2022)												

**Table 1:** Continued.

Author	Year	Type of study	Infected	Joints	PJI	Special condition	WBC	Sensitivity	PMN	Sensitivity	Cutoff	Reporting
			patients/		reference		count	and	cutoff	and	determination	funds
			total		standard		cutoff	specificity		specificity		
Asa Nilsdotter	2007	Prospective	25/85	Hips	Symptoms	No	1700	86 %/92 %	NA	NA	Youden	NM
(Nilsdotter-					Cultures							
Augustinsson et al.,					Sinus							
2007)												
Giovanni Balato (Balato et	2017	Prospective	16/51	Knees	MSIS	No	3000	75 %/91.4 %	80 %	75 %/97 %	Pre-determined	NM
al., 2018b)												
Benjamin Zmistowski	2013	Retrospective	73/150	Knees	Cultures	No	3000	93 %/94 %	75 %	93 %/83 %	Youden	NM
(Zmistowski et al.,					Synovial							
2012)					fluid							
Pieter Berger (Berger et	2017	Prospective	34/121	Hips and	MSIS	No	3000	89.3 %/96.2 %	80 %	89.3 %/92.2 %	Pre-determined	NM
al., 2017)				knees								
Peter N. Chalmers (Chalmers	2015	Prospective	91/433	Knees	Cultures	Fracture	4450	90 %/98.5 %	73 %	89.4 %/93.6 %	Youden	NM
et al., 2014)					Sinus							
					Synovial							
					fluid							
Cara A. Cipriano (Cipriano	2012	Prospective	165/871	Hips and	Cultures	No	3450	91 %/93 %	78 %	95.5 %/87.3 %	Youden	No
et al., 2012)				knees	Synovial	Inflammation	3444	88.2 %/80 %	75 %	100 %/93 %	Youden	funding
					fluid							
Alex Dinnen (Dinneen et	2013	Prospective	34/75	Hips and	Cultures	No	1590	89.5 %/91.3 %	65 %	89.7 %/86.6 %	Youden	NM
al., 2013)			knees	Histology								
				Hips			1425	NA	65 %	NA	Youden	
				Knees			1715	NA	54 %	NA	Youden	
Eli Ghanem	2008	Retrospective	91/128	Knees	Cultures	No	1760	95.6 %/68.6 %	73 %	95.6 %/73 %	Youden	Yes
(Ghanem et al.,					X-rays							
2008b)					Purulence							
					CRP							
Eli Ghanem	2008	Retrospective	161/429	Knees	Abscess	No	1100	90.7 %/88.1 %	64 %	95 %/94.7 %	Youden	Yes
(Ghanem et al.,					Sinus							
2008a)					Cultures							
					Purulence							
Nathanel Heckmann	2023	Retrospective	21/172	Knees	ICM 2018	No	3000	85.7 %/100 %	80 %	79.2 %/99.1 %	Pre-determined	NM
(Heckmann et al.,												
2023)												
Carlos A. Higuera (Higuera et	2017	Retrospective	79/453	Hips	MSIS	No	3966	89.5 %/91.2 %	80 %	92.1 %/85.8 %	NA	No
al., 2017)												funding
Joshua Bingham	2014	Retrospective	19/61	Hips and	MSIS	No	1700	95 %/85 %	NA	NA	Youden	Yes
(Bingham et al.,				knees								
2014)												

**Table 1:** Continued.

Author	Year	Type of study	Infected	Joints	PJI	Special condition	WBC	Sensitivity	PMN	Sensitivity	Cutoff	Reporting
			patients/		reference		count	and	cutoff	and	determination	funds
			total		standard		cutoff	specificity		specificity		
Markus Lenski (Lenski and	2014	Retrospective	31/69	Hips,	MSIS	No	2300	60 %/94.3 %	NA	NA	Youden	NM
Scherer, 2014)				knees,								
				and								
				shoulders								
Young-Min Kwon (Kwon et	2016	Retrospective	7/62	Hips	MSIS	ALTRs	730	86 %/80 %	65 %	100 %/70 %	Youden	No
al., 2016)							892	71 %/80 %	74 %	83 %/70 %	Youden	funding
							2700	71 %/93 %	75 %	83 %/79 %	Youden	
							2930	57 %/93 %	80 %	50 %/79 %	Youden	
							3835	57 %/95 %	84 %	50 %/85 %	Youden	
							4400	43 %/95 %	87 %	33 %/85 %	Youden	
							6800	43 %/96 %	89 %	33 %/89 %	Youden	
							12 600	14 %/96 %	94 %	17 %/94 %	Youden	
J. Bohannon Mason (Mason et	2003	Retrospective	31/86	Knees	Cultures	No	5000	19 %/100 %	80 %	57 %/100 %	Paired	No
al., 2003)					Histology		2500	69 %/98 %	60 %	76 %/89 %	Paired	funding
Mohamed Omar (Omar et al.,	2015	Prospective	21/80	Hips	Sinus	No	3089	85 %/86.3 %	72.1 %	90 %/99 %	Youden	NM
2015)					Purulence							
					Cultures							
Javad Parvizi (Parvizi et	2006	Prospective	94/145	Hips and	Cultures	No	1760	90 %/99 %	73 %	93 %/95 %	Youden	NM
al., 2006)				knees	Purulence							
					CRP/ESR							
Paul H. Yi (Yi et al.,	2015	Retrospective	19/150	Hips	MSIS	ATLRs	4350	100 %/95 %	85 %	82 %/87 %	Youden	Yes
2015)												
Society	2012	Retrospective	28/259	Unicondylar knees	Cultures	No	6200	90 %/96.5 %	60 %	90.8 %/93.8 %	Youden	NM
Unicondylar Knee					Sinus							
(Schwartz et al.,					Purulence							
2012)					Histology							
Ricardo Sousa (Sousa et al.,	2017	Prospective	23/55	Hips and	ICM 2018	No	1463	100 %/71.9 %	78 %	87 %/71.9 %	Youden	No
2017)				knees			2064	91.3 %/75 %	81 %	78.3 %/75 %	Youden	funding
Andrej Trampuz (Trampuz	2004	Prospective	34/133	Knees	Cultures	No	1700	94 %/88 %	65 %	97 %/98 %	NM	Yes
et al., 2004)					Purulence							
					Histology							
Roshan P. Shah (Shah et	2016	Retrospective	14/121	Hips and	MSIS	Fracture	2707	100 %/65 %	77 %	100 %/63 %	Youden	Yes
al., 2016)				knees								

NA means “not available”.

We extracted 135 different cutoffs for synovial WBC count and 114 different cutoffs for synovial PMN. The distribution by joint for synovial WBC count was 63 for hips and knees together, 28 for knees only, 31 for hips only, 4 for shoulders only, and 7 for all four joints together. The distributions by joint for synovial polymorphonuclear proportion (PMN %) were 52, 29, 29, 2, and 4, respectively. There was also one study that focused on unicompartmental knee replacements (Schwartz et al., 2012), one on hip hemiarthroplasties (Salimy et al., 2024), and one on total ankle replacement (Thiesen et al., 2019), which provided information on synovial WBC count and PMN % (Table 2).

**Table 2:** Proposed interpretation cutoffs for joints other than TKA and THA.

	WBC count (cells				
	per microlitre)			PMN (%)	
	Range	Median		Range	Median
Total shoulder	700–12 200	2900		54 %–80 %	67 %
Unicompartmental knee		6200^*^			60 %^*^
Hip hemiarthroplasty		2700^*^			81 %^*^
Total ankle		NA^*^			NA^*^

^*^ Only one paper for this condition. TKA: total knee arthroplasty. THA: total hip arthroplasty. WBC: white blood cell. PMN %: polymorphonuclear proportion. NA: not available.

Considering all papers with no sub-groupings at all, the proposed optimal cutoffs varied widely between 700 and 19 240 cells per microlitre and between 53 % and 99 % PMN. Median values were 3000 cells per microlitre and 75.4 % PMN for all joints and all conditions. Median values for knees, hips, and shoulders separately were 3000 cells per microlitre and 71.5 % PMN, 3000 cells per microlitre and 80 % PMN, and 2900 cells per microlitre and 67 % PMN, respectively.

### 3.4 Meta-analysis

**Figure 3:**
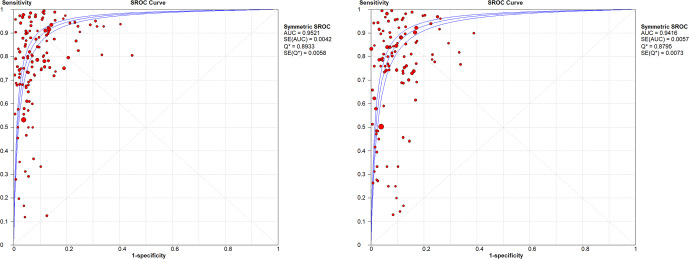
sROC for WBC count and PMN % in all joints.

WBC count showed a summary DOR of 58.38 (95 % CI: 48.48–70.32), an AUC of the sROC of 0.952 (SE: 0.0042), and a Q index of 0.893. PMN proportion showed a summary DOR of 43.17 (95 % CI: 35.31–52.79), an AUC of the sROC of 0.941 (SE: 0.057), and a Q index of 0.879 (Fig. 3).

The heterogeneities ( $I^{2}$ ) of sensitivity and specificity for WBC count were 99.3 % and 88.3 %, respectively, and for PMN % they were 93.9 % and 88.5 %, respectively. DOR heterogeneity was 77.4 % for WBC count and 77.9 % for PMN %.

**Figure 4:**
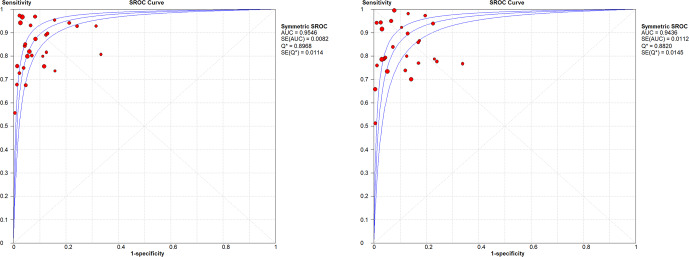
sROC for WBC count and PMN % in knees.

**Figure 5:**
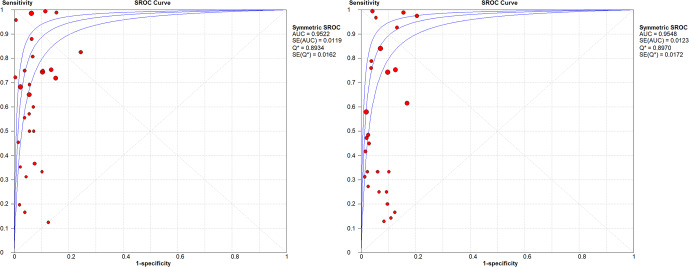
sROC for WBC count and PMN % in hips.

In the sub-group analysis by joint, knees presented a WBC count summary DOR of 83.1 (95 % CI: 54.72–126.21), an AUC of the sROC of 0.954 (SE: 0.0082), a Q index of 0.896, a PMN proportion summary DOR of 63.29 (95 % CI: 38.62–103.74), an AUC of the sROC of 0.936 (SE: 0.011), and a Q index of 0.882. In hips, results showed a WBC count summary DOR of 33.58 (95 % CI: 21.44–52.63), an AUC of the sROC of 0.952 (SE: 0.019), a Q index of 0.893, a PMN proportion summary DOR of 27.12 (95 % CI: 16.22–45.35), an AUC of the sROC of 0.954 (SE: 0.012), and a Q index of 0.897 (Figs. 4 and 5).

**Table 3:** Summary of the proposed cutoffs.

	WBC count (cells per microlitre)				PMN (%)		
	Optimal^1^	Rule-out^2^	Rule-in^3^		Optimal^1^	Rule-out^2^	Rule-in^3^
Chronic PJI^*^	2600	1640	2700		70 %	66.5 %	74.5 %
Hip	2696	–	–		70.5 %	–	–
Knee	2537	–	–		67 %	–	–

^*^ Not enough information available to recommend cutoffs for joints other than TKA and THA. ^1^ Defined by the best combination of sensitivity and specificity. ^2^ Defined by a sensitivity of >95 %. ^3^ Defined by a specificity of >95 %.

### 3.5 Cutoff determination

**Figure 6:**
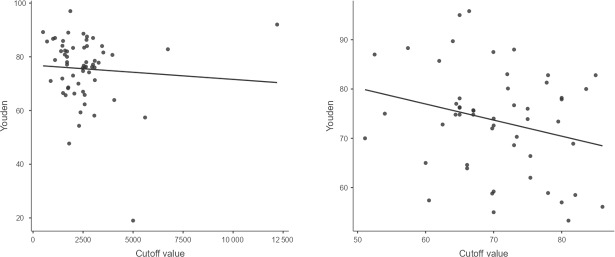
Scatterplot Youden cutoff WBC count and PMN.

In the chronic stage, when no confounding circumstances were present, and excluding papers that used pre-determined cutoffs, the suggested cutoffs varied between 1100 and 5000 cells per microlitre and 53 % and 86 % PMN. The optimal cutoff values were 2537 cells per microlitre for knees, 2696 cells per microlitre for hips, and 70 % PMN for both (Fig. 6, Table 3).

Rule-in cutoffs of ≥2700 cells per microlitre and ≥75 % consistently demonstrated over 95 % specificity, and rule-out cutoffs of ≤1640 cells per microlitre and ≤66 % exceeded 95 % sensitivity for both hips and knees. There was not enough information to recommend a different cutoff for joints other than TKA and THA (Table 3).

When sub-dividing groups between more sensitive PJI definition criteria (ICM, EBJIS, and IDSA; Group 1) and other PJI definition criteria (MSIS and clinical outcome; Group 2), we could see that optimal cutoffs were very similar between them. However, when we applied a rule-in/rule-out calculation, Group 2 presented insufficient data in some cases due to the small number of papers with a specificity or sensitivity of >95 % (Table 4).

**Table 4:** Cutoffs in the modern or more sensitive PJI definition (Group 1) and in the older PJI definition (Group 2).

	WBC count (cells per microlitre)				PMN (%)		
	Optimal^1^	Rule-out^2^	Rule-in^3^		Optimal^1^	Rule-out^2^	Rule-in^3^
Group 1 (ICM, EBJIS, and IDSA)	2530	982	3140		73.7 %	54 %	75.4 %
Group 2 (MSIS and clinical outcome)	2413	1813	2193^*^		68.5 %	66.4 %	69.7 %

^1^ Defined by the best combination of sensitivity and specificity. ^2^ Defined by a sensitivity of >95 %. ^3^ Defined by a specificity of >95 %. ^*^ No papers with >95 % specificity in THA, leaving a reduced sample to compare with other calculations.

### 3.6 Confounding conditions

Some papers were focused on specific conditions that can modify synovial cellularity and PMN %, the so-called confounding conditions. We found six different conditions: five papers were specific to peri-prosthetic fracture associated with PJI (Chalmers et al., 2014; van den Kieboom et al., 2021; Lazic et al., 2022a, b; Shah et al., 2016), six papers to inflammatory conditions (Cipriano et al., 2012; Qin et al., 2022; Ren et al., 2022; Shohat et al., 2018; Tahta et al., 2019; Wang et al., 2023), four papers to adverse local tissue reactions due to head–neck taper corrosion or metal-on-metal bearings (Kwon et al., 2016, 2020; Theil et al., 2024; Yi et al., 2015), one paper to immunosuppressed patients (Lazarides et al., 2019), and one paper to antibiotic use (Salimy et al., 2023).

**Figure 7:**
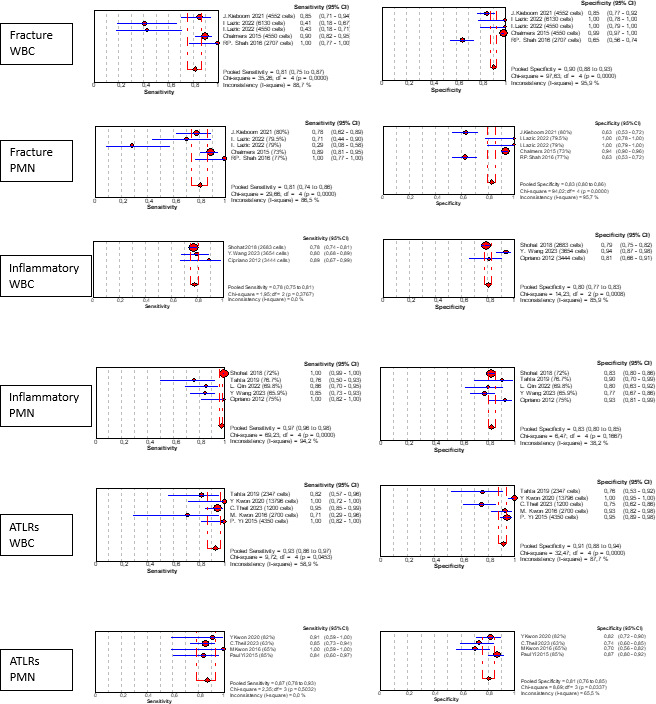
Sensitivity and specificity for special conditions.

The cutoff variability and medians of confounding conditions are presented in Table 5. We were not able to replicate our cutoff interpretation strategy due to the small sample size, but Fig. 7 shows a rough estimate of the diagnostic accuracy in each scenario.

**Table 5:** Summary findings of confounding conditions.

	WBC count (cells per microlitre)			PMN (%)	
	Range	Median		Range	Median
Fracture	2707–6130	4500		73 %–80 %	79.3 %
Inflammatory	1948–5208	3222		65.9 %–85.3 %	75 %
ALTRs and metal-on-metal	730–13 796	2815		63 %–94 %	82 %
Immunosuppressed		1293^*^			
Antibiotic		2339^*^			83 %^*^

^*^ Only one paper for this condition. WBC: white blood cell. PMN %: polymorphonuclear proportion. ALTRs: adverse local tissue reactions.

## 4 Discussion

Accurate PJI diagnosis is challenging, as there is no perfect gold standard test. Definitive diagnosis must therefore rely on a set of pre-determined criteria that include both preoperative and intraoperative findings such as microbiological and histological results. In the past decade, several different PJI definitions have been proposed, which have all included synovial fluid examination (Mcnally et al., 2021; Osmon et al., 2013; Shohat et al., 2019; Workgroup Convened by the Musculoskeletal Infection Society, 2011). Although there is no universally accepted algorithm for diagnosis, it is indisputable that joint aspiration with a differential synovial fluid WBC count is a critical part of current diagnosis.

Our systematic review confirms its value. The DOR and sROC results obtained in our meta-analysis demonstrated that both tests have a high diagnostic accuracy and are powerful tools for reaching PJI diagnosis. On the basis of our results, WBC count analysis demonstrated the highest DOR and sROC values compared with those for PMN proportion. In addition to the high diagnostic accuracy of synovial fluid analysis, it is both inexpensive and widely available. We believe it should therefore continue to be a critical part of any diagnostic flowchart and definition criteria.

Still, there are a number of open questions that we were not able to address entirely in this paper. First, the role of synovial WBC and PMN proportion analysis in the immediate postoperative period is not as well studied. After joint replacement surgery, there is a rise in both WBC count and PMN proportion that takes several weeks to normalize despite an uneventful course (Christensen et al., 2013). We found a number of studies that focused on acute postoperative PJI, but they lacked consistency in how to define infection or even in what timing constitutes the acute period, and therefore we chose to analyse them separately in a different paper.

A different controversy that we were not able to clarify completely is whether the affected joint has an effect on the optimal threshold of the WBC count and/or PMN proportion. The vast majority of papers we found focused on TKA and/or THA separately or combined. We did not find significant differences to recommend adopting different thresholds for hips and knees. In addition, there is not enough available evidence to recommend alternative cutoffs for joints other than TKA and THA.

Though it is not controversial that total WBC count and PMN proportion are highly valuable tests in the diagnostic workup for chronic PJI, still lacking is a universal consensus on what the ideal interpretation thresholds are. Many of the papers that examine the performance of these tests simply adopt previously proposed cutoffs and report on their accuracy in a specific cohort. Many others analyse their own population, adopt certain diagnostic criteria to serve as the PJI gold standard, and offer optimal cutoffs for interpretation. These results are influenced by variables such as population characteristics (e.g. PJI prevalence), a PJI definition standard, or even different laboratory (e.g. microbiology) standards that greatly influence final results. That being said, despite the wide range of proposed cutoffs, we were able to find some consistent results.

The optimal threshold, defined as the median of proposed cutoffs based on the best combination of specificity and sensitivity, was found to be around 2600 cells per microlitre and 70 % PMN overall. Nevertheless, we aimed to find a rule-in threshold by focusing on optimizing specificity at >95 % and found that it was slightly higher (both for WBC count and PMN %). We recommend the 3000 cells per microlitre (sensitivity 88.7 %, specificity 98.5 %) and 75 % PMN (sensitivity 77 %, specificity 97.8 %) thresholds to support the infection diagnosis and definition, as these figures are easy to recall and are compatible with both previously proposed cutoffs and results specifically in papers that use more sensitive or modern definitions (i.e. EBJIS 2021, ICM 2018, and IDSA). On the other hand, when one is looking to rule out PJI during the diagnostic workup, we recommend adopting a lower threshold that optimizes sensitivity at >95 %. Following similar principles enunciated previously, we recommend that infection be suspected and other diagnostic test(s) performed with a WBC count of over 1500 cells per microlitre (sensitivity 100 %, specificity 88 %) and 65 % PMN (sensitivity 95.8 %, specificity 78.6 %).

Lastly, it is crucial to acknowledge that there are special confounding circumstances that hamper the diagnostic accuracy of these tests. Previous antibiotic therapy is associated with a high number of false negative results (Salimy et al., 2023; Shahi et al., 2015; Trampuz et al., 2007). In the chronic setting, this limitation is simple to overcome by withholding antibiotics and repeating the joint tap at a later stage (at least 2 weeks after antibiotic discontinuation). Other pro-inflammatory conditions are perennial and may cause elevated WBC counts regardless of the presence of infection. These circumstances may be joint- or implant-related, e.g. associated peri-prosthetic fractures, dislocation, or metallosis, or may include systemic diseases such as inflammatory arthritis or crystal arthropathy. We found no evidence to justify adopting different condition-specific cutoffs, but information given by the WBC count and/or PMN proportion should be interpreted with caution, as diagnostic accuracy is inferior in these settings.

## 5 Conclusion

Synovial fluid WBC count is a crucial part of any PJI diagnostic workup and definition. In the absence of confounding circumstances, WBC counts of <1500 and >3000 cells per microlitre and proportions of PMN < 65 % and >75 % seem to have very good negative and positive predictive values, respectively. A uniform PJI diagnostic standard is required to facilitate studies able to address persistently open questions, such as the true optimal interpretation thresholds or the need for joint-specific or even condition-specific cutoffs.

## Supplement

10.5194/jbji-10-165-2025-supplement The supplement related to this article is available online at https://doi.org/10.5194/jbji-10-165-2025-supplement.

## Supplement

10.5194/jbji-10-165-2025-supplement
10.5194/jbji-10-165-2025-supplement The supplement related to this article is available online at https://doi.org/10.5194/jbji-10-165-2025-supplement.

## Data Availability

Software code and data supporting the conclusions of this article will be made available by the authors on request.

## Appendix

The supplement related to this article is available online at https://doi.org/10.5194/jbji-10-165-2025-supplement.

## Team list

Marjan Wouthuyzen-Bakker (Department of Medical Microbiology and Infection Prevention, University Medical Center Groningen, University of Groningen, Groningen, the Netherlands), Thomas Bauer (Department of Pathology, The Cleveland Clinic Foundation, Cleveland, OH, USA), Elie Berbari (Division of Public Health, Infectious Diseases and Occupational Medicine, Department of Medicine, Mayo Clinic College of Medicine and Science, Mayo Clinic, Rochester, MN, USA), Martin Clauss (Department of Orthopaedics and Trauma Surgery, University Hospital Basel, Basel, Switzerland), Nicolas Cortes-Penfield (Division of Infectious Diseases, University of Nebraska Medical Center, Omaha, NE, USA), Matthew Dietz (Department of Orthopaedics, School of Medicine, West Virginia University, Morgantown, WV, USA), Jaime Esteban (Department of Medical Microbiology, IIS-Fundacion Jimenez Diaz, Madrid, Spain), Tristan Ferry (Infectious Diseases Department, Croix-Rousse Hospital, Hospices Civils de Lyon, Lyon, France), Thorsten Gehrke (Department of Orthopaedic Surgery, Endo-Klinik Hamburg, Specialist Clinic for Bone and Joint Surgery, Hamburg, Germany), Andor Glaudemans (Medical Imaging Center, Department of Nuclear Medicine & Molecular Imaging, University Medical Center Groningen, University of Groningen, Groningen, the Netherlands), Benjamin Langworth (Division of Biostatistics and Health Data Science, School of Public Health, University of Minnesota, Rochester, MN, USA), Martin McNally (The Bone Infection Unit, Nuffield Orthopaedic Centre, Oxford University Hospitals, Oxford, UK), Andy Miller (Department of Infectious Disease, Hospital for Special Surgery, New York, NY, USA), Sandra Nelson (Division of Infectious Diseases, Massachusetts General Hospital, Harvard Medical School, Boston, MA, USA), Javad Parvizi (Department of Orthopaedic Surgery, International Joint Center, Acibadem University Hospital, Istanbul, Türkiye), Robin Patel (Division of Infectious Diseases, Department of Internal Medicine, Mayo Clinic, Rochester, MN, USA), Holger Rohde (Institute of Medical Microbiology, Virology and Hygiene, University Medical Center Hamburg-Eppendorf, Hamburg, Germany), Thorsten Seyler (Department of Orthopaedics, Duke University Hospital, Durham, NC, USA), Irene Sigmund (Department of Orthopaedics and Trauma Surgery, Medical University of Vienna, Vienna, Austria), Alex Soriano (Department of Infectious Diseases, University of Barcelona, Institut d'Investigacions Biomèdiques August Pi i Sunyer, Hospital Clinic of Barcelona, CIBERINF Ciber in Infectious Diseases, Barcelona, Spain), and Ricardo Sousa (Department of Orthopedics, Centro Hospitalar Universitário de Santo António, Porto, Portugal) on behalf of the Leukocyte Count Synovial Fluid working group for the Unified PJI definition task force.
