# Supplementary material for: Differential synovial fluid white blood cell count for the diagnosis of chronic peri-prosthetic joint infection – a systematic review and meta-analysis
Source: J Bone Jt Infect. 2025 May 14;10(3):165–84. doi: 10.5194/jbji-10-165-2025 (PMC12082335; doi:10.5194/jbji-10-165-2025)
Supplement: The supplement related to this article is available online at https://doi.org/10.5194/jbji-10-165-2025-supplement. [file jbji-10-165-2025-supplement.pdf]

Supplement of J. Bone Joint Infect., 10, 165–184, 2025
https://doi.org/10.5194/jbji-10-165-2025-supplement
© Author(s) 2025. CC BY 4.0 License.

JBJI
Journal of Bone and Joint Infection
Open Access

Supplement of

Differential synovial fluid white blood cell count for the diagnosis of chronic peri-prosthetic joint infection – a systematic review and meta-analysis

Marta Sabater-Martos et al.

Correspondence to: Marta Sabater-Martos (sabaterms@gmail.com)

The copyright of individual parts of the supplement might differ from the article licence.

Table S1: Search strategy

**PubMed, MEDLINE:**
("Arthroplasty"[Mesh] **OR** "Joint Prosthesis"[Mesh] **OR** “Periprosthetic joint infection*”[tiab] **OR** “Prosthetic joint infection*”[tiab] OR “PJI”[tiab] **OR “**Arthroplast*”[tiab] **OR** “Total joint*”[tiab] **OR** “Joint prosthe*”[tiab] **OR** “DAIR”[tiab] **OR** “debridement*”[tiab])<br>**AND**<br>(“cytology” [tiab] **OR** “leucocytes” [tiab] **OR** “neutrophils” [tiab] **OR** “polymor*” [tiab] OR “white blood*” **OR** “lymphocytes” [tiab] **OR** "Leukocytes"[Mesh] **OR** "Neutrophils"[Mesh])<br>**AND**<br>("Sensitivity and Specificity"[Mesh] **OR** “sensitiv*”[tiab] OR “specificit*”[tiab] **OR** “accura*”[tiab] **OR “**positive predictive value*”[tiab] **OR** “negative predictive value*”[tiab] **OR** “PPV” [tiab] **OR** “NPV” [tiab] **OR** “diagnos*” [tiab])
**Embase, EMTREE:**
('arthroplasty'/exp OR 'joint prosthesis'/exp OR 'periprosthetic joint infection'/exp OR ‘Periprosthetic joint infection’:ab,ti,kw OR ‘Prosthetic joint infection*’:ab,ti,kw OR PJI:ab,ti,kw OR Arthroplast*:ab,ti,kw OR ‘Total joint’:ab,ti,kw OR ‘joint prosthe*’:ab,ti,kw OR DAIR:ab,ti,kw OR debridement*:ab,ti,kw)<br>**AND**<br>(‘cytology’:ab,ti,kw **OR** ‘leucocytes’:ab,ti,kw **OR** ‘neutrophils’:ab,ti,kw **OR** ‘polymor*’:ab,ti,kw **OR** ‘white blood*’:ab,ti,kw **OR** ‘lymphocytes’:ab,ti,kw **OR** ‘leukocyte’/exp **OR** ‘synovial fluid’/exp)<br>**AND**<br>('sensitivity and specificity'/exp OR sensitiv*:ab,ti,kw OR specificit*:ab,ti,kw OR accura*:ab,ti,kw OR ‘positive predictive value*’:ab,ti,kw OR ‘negative predictive value*’:ab,ti,kw OR PPV:ab,ti,kw OR NPV:ab,ti,kw) NOT 'conference abstract'/
